# No Evidence of Temporal Decline in Semen Parameters Over 17 Years Among Men Who Underwent Fertility Evaluation from Indian Southern States

**DOI:** 10.1177/15579883251383438

**Published:** 2025-10-21

**Authors:** Huidrom Yaiphaba Meitei, Nithyashree Kadiregowda, Dhakshanya Predheepan, Vani R Lakshmi, Shubhashree Uppangala, Padmaraj Hegde, Guruprasad Kalthur, Stefan Schlatt, Satish Kumar Adiga

**Affiliations:** 1 Centre of Excellence in Clinical Embryology, Department of Reproductive Science, Kasturba Medical College, Manipal, Manipal Academy of Higher Education, Manipal, India; 2 SAR Healthline, Private Ltd., Chennai, India; 3 Department of Health Technology and Informatics, Prasanna School of Public Health, Manipal Academy of Higher Education, Manipal, India; 4 Division of Reproductive Genetics, Department of Reproductive Science, Kasturba Medical College, Manipal, Manipal Academy of Higher Education, Manipal, India; 5 Department of Urology, Kasturba Medical College, Manipal, Manipal Academy of Higher Education, Manipal, India; 6 Division of Reproductive Biology, Department of Reproductive Science, Kasturba Medical College, Manipal, Manipal Academy of Higher Education, Manipal, India; 7 Centre for Reproductive Medicine and Andrology (CeRA), University of Münster, Münster, Germany

**Keywords:** andrology, India, male infertility, semen analysis, sperm quality, spermatozoa

## Abstract

Over the past four decades, reports have emphasized the spatial and temporal trends in human semen quality and other markers of male reproductive health, which have sparked significant debate among fertility professionals. This trend appears limited to specific geographical regions rather than being global. The study aimed to retrospectively review the comprehensive distribution of semen characteristics and analyze their temporal changes over a period of 17 years at a single center in the southern parts of India. Ejaculates of 12,151 sub-fertile men undergoing infertility workup at the University Andrology Laboratory from 2006 to 2022 were included. Semen analysis was performed as per the World Health Organization (WHO, 1999, 2010) recommendations. The primary outcome of interest was temporal changes in semen parameters, whereas data regarding the distribution of semen characteristics were also measured. Sperm concentration, total sperm number, motility, vitality, and morphology showed no significant variations over time. The fifth percentile of semen volume, sperm concentration, total motility, vitality, and normal morphology were 0.5 mL, 1.5 million/mL, 21.0%, 28.0%, and 7.0%, respectively. This study revealed no temporal decline in sperm concentration, motility, and morphology in 17 years, and found fifth percentile sperm characteristics were lower than WHO reference values, marking the extensive long-term semen quality analysis from a single Indian laboratory. The findings reported here do not support an alarming temporal decline in semen quality.

## Introduction

Over the past four decades, numerous reports highlighted the spatio-temporal trends in human semen quality and other male reproductive health markers (Carlsen et al., 1992; De Jonge & Barratt, 2019; De Jonge et al., 2024; Levine et al., 2017). This observation is being reflected in an increasing prevalence of fertility impairment and the number of couples seeking fertility treatment due to suboptimal semen quality (Carson & Kallen, 2021; Eisenberg et al., 2023; Kumar & Singh, 2015; Rama et al., 2023). There is always a question of whether the decline is attributed to economic, environmental, lifestyle, or biological factors (Skakkebæk et al., 2022).

Downward trends in semen quality raised considerable debate among fertility professionals (Jørgensen et al., 2021) and received extensive media coverage, exaggerated by the internet, which might have led to distorted conclusions and undue attention (Auger et al., 2022). Interestingly, the downward trend was found to be limited to specific geographical areas rather than a global phenomenon (Virtanen et al., 2017). A significant decline in sperm number was found in North America, Europe, Australia/New Zealand, whereas too few studies have been published in the rest of America, Asia, and Africa to draw a conclusion about trends in those continents (Levine et al., 2023). Many researchers were skeptical about the heterogeneous nature of the observations, where some studies confirmed a decreasing trend in semen quality, while others did not find the same in some geographical areas (Carlsen et al., 2005; Jørgensen et al., 2012). Studies reported clinically significant changes in the semen profile (Auger et al., 2022; Levine et al., 2017, 2023; Virtanen et al., 2017), whereas other studies concluded that the significant changes observed are mild and may not affect fertility (Feferkorn et al., 2021). Inconsistency in the results led several researchers to study trends in their own countries and specific cohorts of patients.

To our knowledge, long-term studies on semen quality in the Indian population are limited. Previously, we compared the ejaculate quality in 7,700 men from the southern parts of India who attended fertility workup between 1993 and 1994, and 2004 and 2005, and the results showed a significant decline in sperm concentration, motility, and morphology (Adiga et al., 2008). Similarly, another report, which analyzed published data between 1979 and 2016, observed a temporal decline in the semen parameters of Indian men (Mishra et al., 2018).

India, the second most populous country, is influenced by several demographic factors, including diverse backgrounds, varied quality of life, accessibility to health care systems, and different climatic conditions. The fertility rate in India has seen a decline from 4.60 (1980) to 1.91 (2021) and is projected to decline further to 1.29 by 2050 (GBD, 2021 Fertility and Forecasting Collaborators, 2024). The rising infertility rates have seen a proportional increase in fertility care clinics in India (Tholeti et al., 2024). However, the lack of information on the distribution of semen characteristics and reference values among Indian men, and the temporal variation in a specific demography, is an important health issue that suffers from low awareness among the policy makers, patients, researchers, and health care professionals involved in fertility management. With this concern, this study aimed to retrospectively review the comprehensive distribution of semen characteristics of 12,151 sub-fertile men and analyze their temporal changes over a 17-year period at a single infertility center in the southern parts of India, that is, Karnataka, Kerala, and Goa.

## Materials and Methods

This retrospective study includes 21,557 semen analysis reports from 20,000 sub-fertile men who had visited the university infertility center between January 2006 and December 2022 for a fertility checkup. The study was initiated after the approval of the Institutional Ethics Committee (IEC2:198/2023). Data on the patient’s age, abstinence days, number of visits, and semen characteristics were retrieved from each report. The semen characteristics include volume, sperm concentration, motility, vitality, and morphology. Semen analysis was performed as per the World Health Organization (WHO, 1999, 2010) recommendations.

Of 21,557 datapoints, those reports with patients <20 years (*n* = 15) and >50 years (*n* = 207), ejaculatory abstinence of <2 (*n* = 4123) and >7 days (*n* = 995), ejaculates with acidic pH (<7) (*n* = 202), round cells (>1 million/mL) (*n* = 1,300), globozoospermic ejaculates (*n* = 5), reports with presence of abnormal clumping (aggregates and agglutinates) (*n* = 454), pinheaded spermatozoa (*n* = 6), missing values (*n* = 2,321), and ejaculates collected outside the laboratory (*n* = 100) were excluded. A total of 12,151 reports from first-time semen analysis were included for the analysis, of which 862 reports were found azoospermic and hence excluded from the analysis.

Ejaculates were collected through masturbation in a sterile container. The samples were liquified for 30 min at 22°C to 24°C, and semen analysis was initiated within 30 to 60 min after the sample collection. The semen analysis report included data on abstinence days, semen volume, viscosity, sperm concentration, total sperm number, total motility, progressive motility, vitality, and sperm morphology. The pH assessment was assessed using a pH strip (6.5–9.0 range), and the samples were reported as alkaline or acidic. Assessment of semen volume and viscosity was performed using graduated pipettes. Manual assessments were practiced to assess sperm concentration and motility. A Makler counting chamber (Sefi Medical Instruments Ltd., Israel) was used to assess sperm concentration. Sperm motility was performed by manual assessment under 400X magnification with a minimum of 200 cells. The total motility assessment includes the sum of motility patterns grades “a,” “b,” and “c” for the samples included from 2006 to 2010, while the sum of progressive and non-progressive motility comprises total motility for the samples from 2011 to 2022. The sum of motility patterns “a” and “b” had been considered as overall progressive motility for the samples included from 2006 to 2010. The vitality was assessed using eosin–nigrosine across the study periods. The morphology was assessed using the Shorr staining method. Different categories of head defect were scored as per the WHO (1999) fourth edition and the WHO (2010) fifth edition. All technicians involved in the manual assessments underwent standardized training prior to analysis to ensure consistency in the methodology. Inter-observer variability checks were performed on a subset of data, and agreement levels were found to be acceptable.

## Statistical Analysis

Statistical data were analyzed using Jamovi (version 2.3) (Jamovi, 2022). The overall distribution of data was analyzed using the Shapiro–Wilk test. Data are presented as mean and standard deviation, median (interquartile range [IQR]), and frequencies (proportions). The temporal trend of sperm quality across the 17 years was analyzed by either the Kruskal–Wallis test or analysis of variance (ANOVA) based on the normality. *p* < .05 was considered significant at the 5% level of significance.

## Results

### Temporal Trend in Semen Quality

The temporal trend in semen quality is presented in Table 1. About 95% of the patients included in this analysis were from three major states in the southern parts of India, that is, Karnataka, Kerala, and Goa. The mean age range of the study population was 35 to 36 years, which was comparable over time. The mean semen volume and sperm concentration (millions/mL) ranged between 2.4 and 2.6 mL, and 37 and 40 million/mL across the years (Table 1). Both total sperm number and total motility did not show any significant variations over time (103–118 million/ejaculate and 53%–55%, respectively) (Supplementary Fig. 1). The average progressive motility observed over 17 years was 38% to 41%. Similarly, sperm vitality was 56% to 58% across the years, which did not show any significant variation across the study period.

**Table 1.** Temporal Trend in Semen Characteristics Across the Study Period (n = 11,289).

Year of visit	n	Age (years)	Volume (mL)	Sperm concentration (million/mL)	Total sperm number (millions)	Total motility (%)	Progressive motility (%)	Vitality (%)	Morphologically normal spermatozoa (%)
2006	364	35.7 ± 5.3 35.0 (32.0, 39.0)	2.6 ± 1.4 2.5 (1.5, 3.5)	37.4 ± 28.7 35.0 (11.0, 59.2)	107.4 ± 110.7 72.0 (22.5, 156.0)	54.4 ± 16.7 58.0 (45.0, 67.0)	39.8 ± 15.7 42.0 (29.0, 51.0)	57.9 ± 15.2 60.0 (50.0, 70.0)	24.3 ± 12.0 15.0 (23.0, 33.0)
2007	524	36.1 ± 5.1 36.0 (32.7, 40.0)	2.5 ± 1.4 2.5 (1.5, 3.0)	36.4 ± 31.1 31.0 (8.0, 56.0)	103.6 ± 120.5 66.0 (13.9, 152.2)	53.5 ± 17.5 58.0 (44.0, 66.0)	38.3 ± 16.5 41.0 (27.0, 50.0)	56.7 ± 16.7 60.0 (47.0, 69.0)	23.5 ± 11.5 14.0 (22.0, 31.0)
2008	491	36.0 ± 5.1 36.0 (32.0, 40.0)	2.4 ± 1.3 2.5 (1.5, 3.0)	37.7 ± 32.0 29.0 (9.0, 60.0)	103.3 ± 111.8 66.0 (15.0, 153.5)	54.7 ± 16.5 59.0 (44.0, 67.0)	40.2 ± 15.8 42.0 (29.0, 52.0)	57.5 ± 16.0 60.0 (48.0, 70.0)	22.8 ± 11.8 13.0 (21.0, 31.0)
2009	605	36.3 ± 5.0 36.0 (33.0, 40.0)	2.6 ± 1.4 2.5 (1.5, 3.0)	40.3 ± 32.5 35.0 (13.0, 62.0)	118.1 ± 135.6 76.0 (22.5, 168.0)	55.8 ± 17.0 60.0 (47.0, 69.0)	40.5 ± 15.9 42.0 (31.0, 52.0)	58.7 ± 16.1 60.0 (50.0, 70.0)	24.1 ± 11.7 15.0 (22.0, 32.0)
2010	617	36.2 ± 4.9 36.0 (33.0, 39.0)	2.5 ± 1.3 2.5 (1.5, 3.0)	37.8 ± 30.8 31.0 (9.0, 60.0)	107.8 ± 122.4 72.0 (16.0, 152.0)	54.3 ± 17.7 59.0 (45.0, 67.0)	39.5 ± 16.5 41.0 (29.0, 52.0)	57.7 ± 15.8 60.0 (48.0, 70.0)	23.4 ± 11.4 14.0 (22.0, 32.0)
2011	684	36.0 ± 5.1 36.0 (32.0, 40.0)	2.6 ± 1.5 2.5 (1.5, 3.5)	40.5 ± 30.7 38.0 (14.0, 61.0)	116.5 ± 125.1 80.0 (23.0, 162.0)	55.8 ± 17.1 60.0 (48.0, 68.0)	41.0 ± 15.9 43.0 (32.0, 52.0)	59.2 ± 15.3 62.0 (50.0, 70.0)	23.9 ± 11.8 15.0 (22.0, 31.2)
2012	700	35.9 ± 5.0 35.0 (32.0, 39.0)	2.5 ± 1.3 2.5 (1.5, 3.5)	38.5 ± 30.4 34.0 (11.0, 60.0)	109.5 ± 121.1 75.0 (21.8, 162.0)	54.4 ± 17.2 59.0 (45.0, 67.0)	39.9 ± 16.0 43.0 (29.7, 52.0)	58.2 ± 15.7 60.0 (50.0, 70.0)	23.6 ± 11.7 14.0 (22.0, 32.0)
2013	657	35.7 ± 5.2 35.0 (32.0, 39.0)	2.6 ± 1.5 2.5 (1.5, 3.5)	38.3 ± 31.7 31.0 (11.0, 60.0)	113.1 ± 130.5 70.0 (20.0, 157.5)	54.6 ± 16.7 59.0 (45.0, 67.0)	39.8 ± 16.1 42.0 (28.0, 52.0)	58.1 ± 15.5 60.0 (50.0, 70.0)	24.1 ± 11.8 14.0 (23.0, 33.0)
2014	757	35.9 ± 4.9 35.0 (32.0, 39.0)	2.6 ± 1.5 2.5 (1.5, 3.5)	40.1 ± 31.6 35.0 (12.0, 63.0)	116.0 ± 125.7 75.0 (20.0, 175.0)	55.2 ± 16.7 60.0 (47.0, 67.0)	40.1 ± 16.0 43.0 (29.0, 52.0)	58.9 ± 15.8 60.0 (50.0, 70.0)	24.7 ± 12.5 15.0 (24.0, 34.0)
2015	846	36.0 ± 5.0 35.0 (33.0, 39.0)	2.5 ± 1.3 2.5 (1.5, 3.5)	37.5 ± 30.5 33.0 (11.0, 58.0)	107.2 ± 114.4 73.2 (18.0, 158.6)	54.3 ± 16.9 58.0 (44.0, 66.0)	39.4 ± 16.6 42.0 (28.0, 51.0)	57.7 ± 15.5 60.0 (50.0, 69.0)	23.7 ± 11.9 14.0 (23.0, 32.0)
2016	922	36.1 ± 5.0 36.0 (33.0, 40.0)	2.5 ± 1.4 2.5 (1.5, 3.0)	39.3 ± 32.0 33.5 (10.0, 62.0)	112.0 ± 127.0 70.0 (18.0, 167.2)	54.3 ± 16.8 58.0 (44.0, 67.0)	39.5 ± 16.4 42.0 (28.0, 51.0)	58.5 ± 15.7 60.0 (50.0, 70.0)	23.8 ± 11.7 15.0 (23.0, 32.0)
2017	838	36.2 ± 5.1 36.0 (33.0, 39.0)	2.5 ± 1.4 2.5 (1.5, 3.0)	39.6 ± 31.6 34.0 (11.0, 62.0)	111.9 ± 128.4 78.0 (20.1, 156.0)	54.2 ± 17.2 58.0 (44.0, 67.0)	39.1 ± 16.2 42.0 (27.0, 51.0)	58.5 ± 15.9 60.0 (50.0, 70.0)	23.7 ± 11.3 14.2 (23.0, 31.0)
2018	855	35.7 ± 4.9 35.0 (32.0, 39.0)	2.5 ± 1.4 2.0 (1.5, 3.0)	38.6 ± 31.0 33.0 (11.0, 60.0)	110.5 ± 130.4 75.0 (18.0, 155.5)	54.7 ± 16.2 58.0 (45.0, 67.0)	40.2 ± 15.6 43.0 (29.0, 52.0)	58.3 ± 15.5 60.0 (50.0, 70.0)	23.2 ± 12.0 14.0 (22.0, 31.0)
2019	723	36.0 ± 5.0 36.0 (33.0, 39.0)	2.5 ± 1.4 2.5 (1.5, 3.5)	39.0 ± 30.8 34.0 (12.0, 61.0)	116.3 ± 135.8 72.5 (19.0, 164.0)	54.4 ± 17.1 59.0 (45.0, 67.0)	39.1 ± 16.4 42.0 (28.0, 51.0)	58.4 ± 15.6 60.0 (50.0, 70.0)	24.2 ± 12.3 14.0 (23.0, 32.0)
2020	373	36.0 ± 5.1 36.0 (33.0, 39.0)	2.4 ± 1.3 2.0 (1.5, 3.0)	39.8 ± 32.0 36.0 (12.0, 62.0)	107.0 ± 112.4 70.0 (20.0, 158.0)	54.0 ± 17.3 58.0 (45.0, 67.0)	39.2 ± 16.2 42.0 (26.0, 52.0)	58.7 ± 15.5 61.0 (50.0, 70.0)	23.4 ± 11.5 14.0 (22.0, 32.0)
2021	570	35.8 ± 4.9 36.0 (33.0, 39.0)	2.6 ± 1.3 2.5 (1.5, 3.5)	37.5 ± 30.6 31.0 (11.0, 57.0)	108.5 ± 118.2 70.0 (18.2, 160.0)	54.4 ± 16.6 58.0 (46.0, 66.0)	39.6 ± 15.9 42.0 (29.0, 51.0)	57.7 ± 15.7 60.0 (50.0, 70.0)	23.6 ± 11.4 14.0 (22.5, 31.0)
2022	763	35.9 ± 4.9 35.0 (32.0, 39.0)	2.5 ± 1.4 2.5 (1.5, 3.2)	38.4 ± 30.2 34.0 (10.0, 61.0)	110.8 ± 124.4 70.0 (19.8, 162.2)	54.7 ± 16.9 59.0 (46.0, 67.0)	39.5 ± 16.0 42.0 (29.0, 52.0)	57.8 ± 16.2 60.0 (50.0, 70.0)	23.3 ± 11.7 14.0 (22.0, 32.0)
*p* value		.73	.84	.57	.78	.71	.54	.55	.52

*Note.* Values are in *Mean* ± *SD* and median (Q1, Q3); significant at 5% level of significance based on Kruskal–Wallis ANOVA.

The number of morphologically normal spermatozoa did not show a significant decline across the years (23%–24%) (Table 1). Furthermore, the assessment of specific morphological abnormalities showed a consistent trend throughout the study period (Table 2).

**Table 2.** Temporal Trend in Sperm Morphological Abnormalities (%) Across the Study Period (n = 11,289).

Year of visit	*n*	Small head	Large head	Amorphous head	Pyriform head	Tapered head	Tail defects	Cytoplasmic droplets
2006	364	20.9 ± 12.7 19.0 (12.0, 27.0)	10.7 ± 10.0 9.0 (4.0, 14.0)	16.8 ± 10.1 15.5 (10.0, 22.0)	1.0 ± 1.9 0.0 (0.0, 1.8)	1.4 ± 3.4 0.0 (0.0, 2.0)	21.8 ± 14.1 22.0 (10.7, 32.0)	3.9 ± 5.3 2.0 (0.0, 6.0)
2007	524	19.7 ± 12.7 18.0 (11.0, 26.0)	10.7 ± 10.8 8.0 (4.0, 14.0)	17.6 ± 10.9 16.0 (10.0, 24.0)	1.0 ± 1.8 0.0 (0.0, 1.0)	1.4 ± 2.6 0.0 (0.0, 2.0)	21.2 ± 14.9 21.0 (8.0, 32.0)	3.5 ± 4.7 2.0 (0.0, 5.0)
2008	491	21.7 ± 14.5 20.0 (11.0, 28.0)	11.8 ± 11.2 9.0 (5.0, 15.0)	17.5 ± 11.9 15.0 (9.0, 24.0)	1.1 ± 2.7 0.0 (0.0, 1.0)	1.2 ± 2.5 0.0 (0.0, 2.0)	20.7 ± 14.4 21.0 (9.0, 32.0)	3.2 ± 5.1 1.0 (0.0, 4.0)
2009	605	20.7 ± 13.2 19.0 (12.0, 28.0)	11.1 ± 11.3 8.0 (4.0, 14.0)	16.7 ± 10.9 15.0 (9.0, 22.0)	1.0 ± 2.4 0.0 (0.0, 1.0)	1.5 ± 4.4 0.0 (0.0, 2.0)	21.1 ± 14.4 21.0 (9.0, 32.0)	4.0 ± 6.7 2.0 (0.0, 5.0)
2010	617	21.0 ± 13.5 19.0 (12.0, 28.0)	11.0 ± 10.9 8.0 (4.0, 14.0)	17.8 ± 11.2 16.0 (10.0, 24.0)	0.9 ± 1.9 0.0 (0.0, 1.0)	1.5 ± 3.2 0.0 (0.0, 2.0)	19.6 ± 13.6 20.0 (8.0, 28.0)	3.7 ± 5.8 2.0 (0.0, 5.0)
2011	684	21.0 ± 14.3 19.0 (12.0, 26.0)	11.4 ± 11.6 8.0 (4.0, 14.0)	16.2 ± 10.9 15.0 (8.0, 23.0)	0.9 ± 2.0 0.0 (0.0, 1.0)	1.5 ± 3.1 0.0 (0.0, 2.0)	20.2 ± 14.6 20.0 (7.0, 30.0)	3.6 ± 5.0 2.0 (0.0, 5.0)
2012	700	20.3 ± 13.0 18.0 (11.0, 27.0)	11.6 ± 11.9 8.0 (4.0, 14.0)	17.1 ± 10.9 16.0 (9.0, 23.0)	1.0 ± 1.9 0.0 (0.0, 1.0)	1.3 ± 2.8 0.0 (0.0, 2.0)	20.2 ± 14.4 20.0 (8.0, 32.0)	3.2 ± 4.9 2.0 (0.0, 5.0)
2013	657	20.1 ± 12.6 18.0 (12.0, 26.0)	10.9 ± 10.7 8.0 (4.0, 14.0)	17.7 ± 11.3 16.0 (10.0, 23.0)	1.0 ± 2.0 0.0 (0.0, 1.0)	1.1 ± 2.3 0.0 (0.0, 2.0)	20.4 ± 14.4 20.0 (8.0, 31.0)	3.3 ± 4.6 2.0 (0.0, 5.0)
2014	757	21.0 ± 13.8 19.0 (12.0, 28.0)	10.6 ± 10.9 8.0 (4.0, 13.0)	16.9 ± 11.4 15.0 (9.0, 24.0)	0.9 ± 1.7 0.0 (0.0, 1.0)	1.5 ± 3.7 0.0 (0.0, 2.0)	19.5 ± 13.7 20.0 (8.0, 29.0)	3.6 ± 5.5 2.0 (0.0, 5.0)
2015	846	20.8 ± 13.2 19.0 (12.0, 27.7)	10.1 ± 9.6 8.0 (4.0, 13.0)	17.3 ± 11.1 16.0 (9.0, 24.0)	1.0 ± 2.3 0.0 (0.0, 1.0)	1.3 ± 3.0 0.0 (0.0, 2.0)	20.7 ± 14.5 20.5 (8.2, 31.0)	4.0 ± 5.4 2.0 (0.0, 6.0)
2016	922	20.7 ± 13.2 19.0 (12.0, 27.0)	11.4 ± 11.2 8.0 (4.0, 15.0)	16.9 ± 11.4 15.0 (8.0, 23.0)	1.0 ± 2.5 0.0 (0.0, 1.0)	1.5 ± 3.3 0.0 (0.0, 2.0)	20.0 ± 14.2 20.0 (8.0, 30.0)	3.6 ± 5.6 2.0 (0.0, 5.0)
2017	838	21.0 ± 14.2 18.0 (12.0, 27.0)	11.1 ± 11.2 8.0 (4.0, 14.0)	17.1 ± 11.2 16.0 (9.0, 23.0)	1.0 ± 2.2 0.0 (0.0, 1.0)	1.3 ± 2.9 0.0 (0.0, 2.0)	20.3 ± 13.9 21.0 (8.0, 31.0)	3.7 ± 5.4 2.0 (0.0, 5.0)
2018	855	20.3 ± 13.6 18.0 (12.0, 28.0)	11.0 ± 11.0 8.0 (4.0, 14.0)	17.3 ± 11.2 16.0 (9.0, 24.0)	1.1 ± 2.1 0.0 (0.0, 2.0)	1.4 ± 3.1 0.0 (0.0, 2.0)	20.3 ± 14.7 20.0 (8.0, 31.0)	3.7 ± 5.5 2.0 (0.0, 5.0)
2019	723	20.2 ± 13.5 18.0 (11.0, 27.0)	11.8 ± 12.0 8.0 (4.0, 15.0)	16.8 ± 11.2 15.0 (9.0, 23.0)	1.0 ± 1.9 0.0 (0.0, 2.0)	1.4 ± 3.4 0.0 (0.0, 2.0)	20.3 ± 14.3 20.0 (8.0, 30.0)	3.8 ± 6.2 2.0 (0.0, 6.0)
2020	373	20.2 ± 12.4 18.0 (12.0, 28.0)	10.4 ± 10.6 8.0 (4.0, 13.0)	18.0 ± 10.8 17.0 (10.0, 24.0)	1.1 ± 3.0 0.0 (0.0, 1.0)	1.2 ± 2.2 0.0 (0.0, 2.0)	22.2 ± 15.0 21.0 (11.0, 32.0)	3.9 ± 5.5 2.0 (0.0, 6.0)
2021	570	19.6 ± 12.6 18.0 (12.0, 25.0)	9.9 ± 10.1 8.0 (4.0, 13.0)	17.7 ± 11.2 17.0 (9.2, 24.0)	1.0 ± 2.0 0.0 (0.0, 1.0)	1.4 ± 2.8 0.0 (0.0, 2.0)	20.8 ± 13.7 21.0 (11.0, 30.0)	3.4 ± 4.7 2.0 (0.0, 5.0)
2022	763	20.6 ± 14.4 18.0 (11.0, 28.0)	11.3 ± 11.4 9.0 (4.0, 15.0)	16.4 ± 11.1 16.0 (9.0, 22.0)	0.9 ± 1.8 0.0 (0.0, 1.0)	1.5 ± 4.0 0.0 (0.0, 2.0)	20.2 ± 15.4 20.0 (6.0, 32.0)	3.2 ± 4.4 2.0 (0.0, 5.0)
*p* value		.57	.07	.25	.94	.77	.23	.08

*Note.* Values are in *Mean* ± *SD* and median (Q1, Q3); significant at 5% level of significance based on Kruskal–Wallis ANOVA.

Throughout the study period, the prevalence of oligozoospermia ranged between 26% (*n* = 178/684) and 37% (*n* = 194/524), remaining consistent over the years. Similarly, the prevalence of azoospermia was 4% to 11% (*n* = 38/876–79/696) during the study period (Supplementary Table 1), and specifically, about 77% of azoospermic men had non-obstructive pathology (*n* = 664/862). Overall, the prevalence of abnormal semen parameters across the years was 57.1% (*n* = 6,942/12,151).

### Temporal Distribution of Semen Characteristics

The average age of the study subjects included was 36.0 ± 5.0 years (IQR = 32.0, 39.0 years), and they had the ejaculatory abstinence of 4.0 ± 1.5 days (IQR = 3.0, 5.0 days). The average semen volume, sperm concentration, total motility, vitality, and morphologically spermatozoa were 2.5 ± 1.4 mL (IQR = 1.5, 3.5 mL), 38.7 ± 31.1 million/mL (IQR = 11.0, 60.0 million/mL), 54.6 ± 16.9% (IQR = 45.0, 67.0%), 58.2 ± 15.7% (IQR = 50.0, 70.0%), and 23.7 ± 11.8% (IQR = 14.0, 32.0%), respectively (Table 3). The fifth percentile of the sperm characteristics observed in the present study population was lower than the corresponding WHO reference. Specifically, the fifth percentile of semen volume, sperm concentration, total motility, vitality, and normal morphology were 0.5 mL, 1.5 million/mL, 21.0%, 28.0%, and 7.0%, respectively (Table 3).

**Table 3:** Distribution of Semen Characteristics in Men Attending Fertility Workup Between 2006 and 2022 (n = 11,289).

			Percentiles								
	*Mean* ± *SD*	95% CI	2.5th	5th	10 th	25th	50th	75th	90th	95th	97th
Age (years)	36.0 ± 5.0	35.9–36.1	27.0	28.0	30.0	32.0	36.0	39.0	43.0	45.0	47.0
Abstinence (days)	4.0 ± 1.5	3.9–4.0	2.0	2.0	2.0	3.0	4.0	5.0	7.0	7.0	7.0
Semen volume (mL)	2.5 ± 1.4	2.5–2.6	0.5	0.5	1.0	1.5	2.5	3.5	4.5	5.0	6.0
Sperm concentration (10^6^/mL)	38.7 ± 31.1	38.1–39.3	0.6	1.5	3.0	11.0	33.0	60.0	83.0	97.0	107.0
Total sperm number (10^6^/ ejaculate)	111.0 ± 124.4	108.7–113.3	0.75	1.7	4.2	19.5	72.5	160.0	270.0	351.0	430.0
Total motility (%)	54.6 ± 16.9	54.3–54.9	14.0	21.0	30.0	45.0	59.0	67.0	73.0	77.0	79.0
Progressive motility (%)	39.7 ± 16.1	39.4–40.0	5.0	10.0	16.0	29.0	42.0	52.0	59.0	63.0	67.0
Vitality (%)	58.2 ± 15.7	57.9–58.5	20.0	28.0	36.0	50.0	60.0	70.0	77.0	80.0	83.0
Normal forms (%)	23.7 ± 11.8	23.5–24.0	4.0	7.0	10.0	14.0	23.0	32.0	40.0	45.0	49.0
Small head (%)	20.6 ± 13.5	20.3–20.8	0.0	2.0	6.0	12.0	18.0	27.0	37.0	45.0	57.0
Large head (%)	11.0 ± 11.0	10.8–11.2	0.0	0.0	1.0	4.0	8.0	14.0	23.0	35.0	46.0
Amorphous head (%)	17.1 ± 11.1	16.9–17.3	0.0	0.0	4.0	9.0	16.0	23.0	32.0	38.0	44.0
Pyriform head (%)	1.0 ± 2.1	0.9–1.0	0.0	0.0	0.0	0.0	0.0	1.0	3.0	5.0	7.0
Tapered head (%)	1.4 ± 3.2	1.3–1.5	0.0	0.0	0.0	0.0	0.0	2.0	4.0	6.0	8.0
Tail defects (%)	20.5 ± 14.4	20.2–20.7	0.0	0.0	0.0	8.0	20.0	31.0	40.0	44.0	49.0
Cytoplasmic droplets (%)	3.6 ± 5.4	3.5–3.7	0.0	0.0	0.0	0.0	2.0	5.0	10.0	13.0	18.0

## Discussion

This study included 12,151 semen analysis reports from Indian men who underwent fertility evaluation in a single center over a period of 17 years. The data revealed two interesting findings. First, there was no temporal decline in sperm concentration, motility, and morphology in samples collected between 2006 and 2022. Second, the fifth percentile of sperm characteristics in the analyzed ejaculates was lower than the WHO reference values. To our knowledge, this is the most extensive study and is the first to report the long-term semen quality trends within a single laboratory in India.

Temporal changes in semen quality have been reported in many other countries; however, other reports contradict the claim that semen quality has deteriorated. The data regarding temporal trends in sperm quality among the Indian population are limited. The semen profile from various published studies reported a decline in semen quality (Mishra et al., 2018). The unchanged temporal trend observed in this study contradicts our own earlier report, where a significant decline in sperm number and motility was observed in the ejaculate of patients who underwent infertility evaluation between 1996 and 2006 in similar geographical and clinical settings (Adiga et al., 2008). It is possible that the study period or changes in the methods of calculating these values could have contributed to this difference in the trend.

Spatio-temporal variation in semen quality has been widely discussed in the literature (Auger et al., 2022; Levine et al., 2023; Levine et al., 2017; Virtanen et al., 2017). In line with our results, stable semen quality was observed in Argentinian population between 2001 and 2020 (Ramírez et al., 2022), Swedish population between 2000 and 2010 (Axelsson et al., 2011), while recent decline in sperm concentration have been reported in sub-fertile American, Chinese, and population from sub-Saharan countries (Akang et al., 2023; Li et al., 2023; Punjani et al., 2023) and Finnish, Spanish, and French men from the general population (Jørgensen et al., 2011; Mendiola et al., 2013; Rolland et al., 2013). Similarly, a recent meta-analysis performed among the U.S. population suggests no clinically significant decline in sperm concentration in men without a history of infertility (Lewis et al., 2025).

In contrast to prior studies focusing solely on trends in semen quality, we looked at the distribution of semen characteristics. A large number of studies conducted worldwide over the past decades have documented regional variations in semen quality (Auger et al., 2022; Levine et al., 2023; Levine et al., 2017; Virtanen et al., 2017). The sperm concentration observed in our study was lower than the reports from other regions in Asia (Jiang et al., 2014; Nishihama et al., 2017; Palani et al., 2020; Shi et al., 2021; Zhang et al., 2017). In contrast, the total motility in our study was higher than that of sub-fertile men from other regions (Jiang et al., 2014; Najafpour et al., 2020; Nishihama et al., 2017; Shi et al., 2021; Zhang et al., 2017). The discrepancies between our study and previous reports may be due to differences in geographical location, environmental factors, races, and laboratory conditions (Agarwal et al., 2022; Feferkorn et al., 2022; Khandwala et al., 2017; Punjani et al., 2020). It is important to note that the fifth percentile of our study subjects was lower than the WHO reference. Nonetheless, the comparison of the percentiles will be invalid as the populations of our study comprised sub-fertile men, unlike those used to derive WHO reference values.

Technical factors, such as manual assessment for sperm characteristics, might not be the cause of the discrepancies in the results observed. The samples were analyzed by only four technicians with experience of 5 to 15 years, who had been working in the laboratory during the entire study period. The technicians had undergone routine quality control as per the hospital policy. There were no significant changes in the method of analysis across the years. However, implementing the WHO (1999, 2010) fifth edition over the fourth edition was part of the guidelines. The motility categorization across the study period was changed since WHO (2010) recommended to combine rapid and slow progressive motility into progressive motility . To address the potential impact of the transition from the WHO fourth to fifth edition semen analysis guidelines, particularly the change in motility classification from grades a, b, and c to progressive motility, data from both editions were compared using an independent *t*-test to evaluate whether motility parameters differed significantly between samples assessed under the two editions. The analysis showed no statistically significant difference, indicating that the change in classification did not materially affect the results. This supports the comparability of motility data across the study period without the need for additional standardization.

The strength of this study lies in the large number of subjects included, each included only once, and that the study was performed in a single university fertility clinic, thus minimizing confounders by not altering the study population or the analysis. Our study is limited by its retrospective design and by the fact that the study group comprises male partners from infertile couples, which restricts the availability of data on body mass index, cause of infertility, hormonal profile, genetic testing, lifestyle, occupational factors, medications, and other missing reports. The study population may be subject to selection bias, as it comprised male partners referred by primary care physicians, urologists, gynecologists, reproductive endocrinologists, and reproductive medicine specialists. The assessment of sperm concentration by the Makler counting chamber is not in line with the WHO recommendations and might bias the results (Björndahl et al., 2016). While WHO reference values provide a widely used benchmark, they may not fully reflect the characteristics of a sub-fertile population. The lower percentile values observed in our cohort reflect the nature of our study population, which comprised primarily men seeking fertility evaluation. As the study was conducted at a single fertility clinic, the generalizability of our findings to populations from other regions is limited.

It has been suggested that testing a new reagent, different method or procedure for improving on the performance of a current recommendation in andrology should be specified and measured against the recommended methods (Björndahl et al., 2022) hence, the checklist where deviations from the recommended methods were stated is provided in the Supplementary information.

## Conclusion

Our findings suggest that, in contrast to prior reports, semen quality among Indian men from southern states may be more stable and do not support an alarming temporal decline.

## Supplemental Material

sj-docx-1-jmh-10.1177_15579883251383438 – Supplemental material for No Evidence of Temporal Decline in Semen Parameters Over 17 Years Among Men Who Underwent Fertility Evaluation from Indian Southern States Supplemental material, sj-docx-1-jmh-10.1177_15579883251383438 for No Evidence of Temporal Decline in Semen Parameters Over 17 Years Among Men Who Underwent Fertility Evaluation from Indian Southern States by Huidrom Yaiphaba Meitei, Nithyashree Kadiregowda, Dhakshanya Predheepan, Vani R Lakshmi, Shubhashree Uppangala, Padmaraj Hegde, Guruprasad Kalthur, Stefan Schlatt and Satish Kumar Adiga in American Journal of Men's Health

sj-tif-2-jmh-10.1177_15579883251383438 – Supplemental material for No Evidence of Temporal Decline in Semen Parameters Over 17 Years Among Men Who Underwent Fertility Evaluation from Indian Southern States Supplemental material, sj-tif-2-jmh-10.1177_15579883251383438 for No Evidence of Temporal Decline in Semen Parameters Over 17 Years Among Men Who Underwent Fertility Evaluation from Indian Southern States by Huidrom Yaiphaba Meitei, Nithyashree Kadiregowda, Dhakshanya Predheepan, Vani R Lakshmi, Shubhashree Uppangala, Padmaraj Hegde, Guruprasad Kalthur, Stefan Schlatt and Satish Kumar Adiga in American Journal of Men's Health
