# Supplementary material for: No Evidence of Temporal Decline in Semen Parameters Over 17 Years Among Men Who Underwent Fertility Evaluation from Indian Southern States
Source: Am J Mens Health. 2025 Oct 21;19(5):15579883251383438. doi: 10.1177/15579883251383438 (PMC12553896; doi:10.1177/15579883251383438)
Supplement: sj-docx-1-jmh-10.1177_15579883251383438 – Supplemental material for No Evidence of Temporal Decline in Semen Parameters Over 17 Years Among Men Who Underwent Fertility Evaluation from Indian Southern States [file sj-docx-1-jmh-10.1177_15579883251383438.docx]

Supplementary Table 1 – Prevalence of oligozoospermia and azoospermia samples across the study period

| Year | Prevalence (%) of Oligozoospermia | Prevalence (%) of Azoospermia |
| --- | --- | --- |
| 2006 | 34.8 (127/364) | 8.3 (33/397) |
| 2007 | 37.0 (194/524) | 8.2 (47/571) |
| 2008 | 36.6 (180/491) | 8.7 (47/538) |
| 2009 | 33.3 (202/605) | 8.3 (55/660) |
| 2010 | 36.3 (224/617) | 11.3 (79/696) |
| 2011 | 26.0 (178/684) | 8.8 (66/750) |
| 2012 | 29.5 (207/700) | 8.2 (63/763) |
| 2013 | 29.8 (196/657) | 7.4 (53/710) |
| 2014 | 29.0 (220/757) | 5.9 (48/805) |
| 2015 | 30.0 (254/846) | 5.1 (46/892) |
| 2016 | 30.1 (278/922) | 6.5 (65/987) |
| 2017 | 28.5 (239/838) | 4.3 (38/876) |
| 2018 | 30.1 (258/855) | 5.8 (53/908) |
| 2019 | 28.7 (208/723) | 7.5 (59/782) |
| 2020 | 29.4 (110/373) | 4.6 (18/391) |
| 2021 | 28.5 (163/570) | 5.1 (31/601) |
| 2022 | 28.9 (221/763) | 5.0 (41/804) |
| p-value | 0.45 | 0.45 |

*Note: Values are expressed in percentage;* *Significant at 5% level of significance based on Kruskal-Wallis ANOVA*
