# Supplementary figures and images for: No Evidence of Temporal Decline in Semen Parameters Over 17 Years Among Men Who Underwent Fertility Evaluation from Indian Southern States
Source: Am J Mens Health. 2025 Oct 21;19(5):15579883251383438. doi: 10.1177/15579883251383438 (PMC12553896; doi:10.1177/15579883251383438)

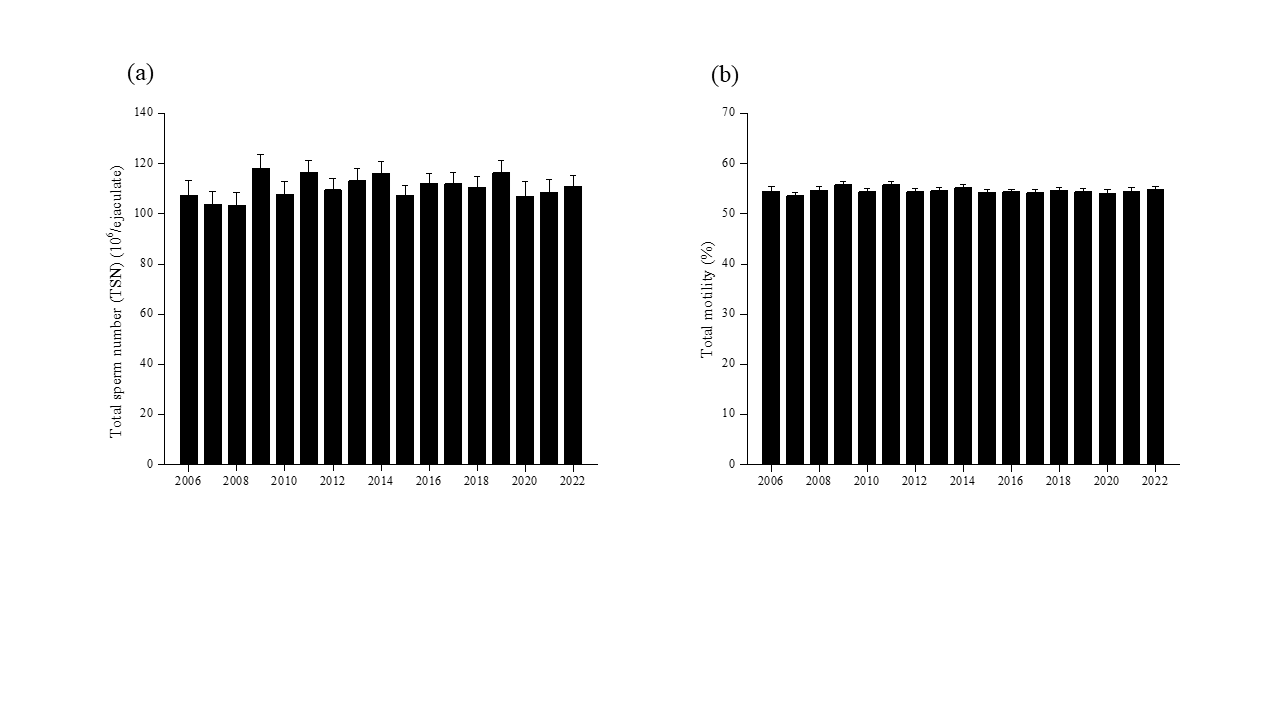

Supplement: sj-tif-2-jmh-10.1177_15579883251383438 – Supplemental material for No Evidence of Temporal Decline in Semen Parameters Over 17 Years Among Men Who Underwent Fertility Evaluation from Indian Southern States [file sj-tif-2-jmh-10.1177_15579883251383438.tif]
